# A stochastic generative model of the World Trade Network

**DOI:** 10.1038/s41598-019-54979-1

**Published:** 2019-12-06

**Authors:** Javier García-Algarra, Mary Luz Mouronte-López, Javier Galeano

**Affiliations:** 1 Department of Engineering, Centro Universitario de Tecnología y Arte Digital, Las Rozas, Spain; 2 Higher Polytechnic School, Universidad Francisco de Vitoria, Pozuelo de Alarcón, Spain; 3 Complex Systems Group, Universidad Politécnica de Madrid, Madrid, Spain

**Keywords:** Applied mathematics, Complex networks

## Abstract

The World Trade Network (WTN) is a network of exchange flows among countries whose topological and statistical properties are a valuable source of information. Degree and strength (weighted degree) are key magnitudes to understand its structure and generative mechanisms. In this work, we describe a stochastic model that yields synthetic networks that closely mimic the properties of annual empirical data. The model combines two popular mechanisms of network generation: preferential attachment and multiplicative process. Agreement between empirical and synthetic networks is checked using the available series from 1962 to 2017.

## Introduction

International trade is the circulatory system of global economy. The flow of goods and services may be modelled as a bipartite network with two classes on nodes: exporters and importers. These nodes are tied by the existence and intensity of exchanges.

The study of the statistical and topological properties of the global trade as a network has been an active research topic in recent years. In these studies, degree and strength are two key connectivity properties of each node. *Degree* is the number of links to nodes of the opposite class, while *strength* is the sum of weights of those links. For WTN nodes, degree is the number of countries they trade with. Each link is weighted by the trade volume between both nodes. Strength accounts for the total ammount of trade of a particular node and equates the sum of weights ot its links. Probability distributions of degree and strength shed light on the network properties^1^. If the network is scale-free, these distributions follow a power-law^2^, also known as Pareto’s law in Economics. Trying to fit empirical data to a power-law has been a common procedure in network analysis, despite the fact that this behavior is not universal^3^.

The log-normal distribution is a second possible choice in many fields, but specially in Economics^4^. The discussion about the best model has run for a long time, because both may fit the long tail of some empirical series^5^. The hypothesis of log-normality dates back to the end of the XIX^th^ Century, but it was the Dutch astronomer Cornelius Kapteyn who first described its pervasive presence and proposed a generative model^6^. He used the growth of plants and animals to explain the multiplicative process that lies behind the emergence of log-normality. Three decades later, Robert Gibrat wrote about the *law of proportional effect*, that stated that the average and variance of growth rates for many industrial processes are nearly constant^7^ and by virtue of the Central Limit Theorem the distribution tends towards log-normality. Fitting empirical data to a log-normal distribution to test Gibrat’s hypothesis was a recurrent research topic, in special working with the size of firms^8^, despite the fact that “the upper tail resembles the Pareto’s distribution” in the own words of the classical work of Ijiri and Simon^9^. Stanley *et al*. found that the distribution of logarithmic annual growth rates was better described by an exponential^10^.

The discussion about the validity of Gibrat’s law to describe empirical distributions appears in different fields of Economics, with examples supporting^11,12^ and rejecting it^13,14^ on statistical grounds. Depending on the particular series, the study may be inconclusive, as in the particular case of Italian hospitality industry^15^.

The dilemma between power-law and log-normal distribution to describe empirical trade volume series^13^ has also been a recurrent topic of the World Trade Network analysis. WTN degree and strength distributions do not show a power-law pattern (see Supplementary Information, Fig. S1) but over the skewed right tails. So, Garlaschelli and Loffredo showed^16^ that WTN cumulative degree distribution is not a power-law as claimed in a precedent work^17^, but that WTN is scale-free using the cumulative GDP distribution as a hidden variable^18^. Batthacarya *et al*. confirmed the power-law relationship between trade volume and GDP while supporting that the strength distribution is log-normal^19^. Kali and Reyes, found that the number of trade relationships among countries follow the 80/20 rule, reproducing at global scale the distribution inequality that was in the basis of Pareto’s original research^20^. Ward *et al*. rejected the degree power-law hypothesis using the gravity model^21^. De Benedictis *et al*., hold the view that the in-degree and out-degree right tail distributions follow a power-law over a wide range of values^22^. On the other hand, log-normality seems a good choice to describe degree and strength distributions for Fagiolo *et al*.^23^ and Barigozzi *et al*.^24^.

Empirical degree or strength data follow neither pure log-normal distributions, nor sharp power-law over all the range of values. A possible explanation is that the statistical properties of the WTN matrixes could be driven by two processes of different nature acting at different time scales, the first driving a fast network build-up, and the second one over a much longer period.

We have designed our model inspired by two precedents, Ijiri and Simon principles to explain log-normality in the size distribution of firms^9^ and the generative approach to a well-known class of bipartite networks in a quite different field, Ecology.

Ijiri and Simon proposed a stochastic model to emulate the size growth of firms using two basic principles: the probability that a new business chance is captured by an existing company is proportional to its size and the probability of arrival of a new company is constant over time. This model was well-suited for a unipartite network that acts on a long-term scale.

In Ecology, mutualistic networks work on the principle of mutual benefit for both classes of nodes, like plants and pollinators. In a different way of human trade they also exchange goods (pollen, nectar) and services (fecundation, protection). For this kind of networks, there has been a long discussion on the best generative model to explain why their degree and strength distributions follow a power-law or a truncated power-law (see a review by Guimaraes *et al*.^25^). Field data sampling in Ecology is a hard task, and empirical data may consist of just one campaign. Generative models in this walk of research try to reproduce the main statistical parameters of the recorded adjacency matrix, from a pure phenomenological point of view. In this work, we adopt the same approach to work with the historical world trade series, with the advantage that the series spans 56 years.

For our study the WTN is a set with two different guilds of nodes: Exporters and Importers. There exists a directed link from country **A** as exporter to country **B** as importer (*Exporter*_*A*_ → *Importer*_*B*_), when *Exporter*_*A*_ sells merchandises to *Importer*_*B*_. The weight of the link is the volume they trade during that year. Any country may be both exporter and importer, so the opposite flow from **B** to **A** happens between a different pair of nodes (*Exporter*_*B*_ → *Importer*_*A*_) and with a different weight. Exporter nodes may link to any Importer, but never among them (and viceversa), and the weight of the relationship is the trade volume flowing from the Exporter to the Importer. Under these circumstances, the WTN is a weighted bipartite network^26^. The adjacency matrix has as many rows as exporters and as many columns as importers. There is one empirical matrix per year that stores the flow from *Exporter*_*i*_ to *Importer*_*j*_ ∀ *i*, *j*. The number of nodes and links changes from year to year, due to geopolitical events and the non-stationary nature of trade itself.

Our approach takes into account two observations: dominant countries, both as exporters or importers, attract with high probability new trade chances and global yearly degree and volume trade distributions are approximately log-normal. In this paper, we describe a generative stochastic synthetic model of trade (*Synthrade*) that mixes two processes, preferential attachment and proportional effect, to mimic the properties of the weighted matrixes of WTN historical data series. The first one works during a very short period of simulation time and yields a scale-free heterogeneous distribution of trade volume for exporter and importer guilds. The second, acts on a much larger time scale and by its multiplicative effect makes the yearly trade distributions log-normal.

The term *growth* is pervasive in Economics and Network Science, but not always with the same meaning. In this paper it makes reference to both possible acceptations, it is mandatory to explain how we use it. When Gibrat writes about firms’ growth, it is clear that he refers to the yearly increase of some proxy variable of its size, like income or number of employees. If a researcher analyzes the world trade volume evolution over the last century, growth means a rate of change of that magnitude. On the other hand, a network is usually a dynamic entity that experiments growth, by the addition of new nodes, links or the increase of the weight of links. When dealing with the synthetic World Trade Network model, network growth is not the same than growth rate of trade volume. In fact, the model does not deal with the second meaning, but just with network growth.

## Results

### Model

The model of the international trade network is created from scratch and computed year by year. Nodes arrive during a very short period of simulation time, at a decreasing pace, and they attach to nodes of the opposite guild with a probability that is proportional to the volume traded by those countries.

An important difference with Ijiri and Simon approach is that we do not try to model trade volume evolution over a long span of time but just the statistical properties of each annual empirical data, using as parameters just three data of the original set as we explain in this section.

A weighted bipartite network ${{\bf{W}}}_{EI}^{i,j}(t)$, represents the trade, where the value of each cell *i*, *j* is the aggregated volume between *Exporter*_*i*_ and *Importer*_*j*_ at simulation instant *t*. The strength of *Exporter*_*i*_ is defined as ${{\bf{S}}}_{E}^{i}(t)={\sum }_{j}\,{{\bf{W}}}_{EI}^{i,j}(t)$. The total trade volume of the synthetic network at instat *t* is the sum ${\sum }_{i,j}\,{{\bf{W}}}_{EI}^{i,j}(t)$ and we call it *global strength*.

There is another matrix $${{\bf{P}}}_{EI}^{i,j}(t)$$, that accounts for the probability of trade among countries. At each simulation step *t*, $${{\bf{P}}}_{EI}^{i,j}(t)$$ is the outer product of $${{\bf{P}}}_{E}^{i}(t-1)$$ and $${{\bf{P}}}_{I}^{j}(t-1)$$, being $${{\bf{P}}}_{E}^{i}(t-1)=\frac{{{\bf{S}}}_{E}^{i}(t-1)}{{\sum }_{i}\,{{\bf{S}}}_{E}^{i}(t-1)}$$, $${{\bf{P}}}_{I}^{j}(t-1)=$$$$\frac{{{\bf{S}}}_{I}^{j}(t-1)}{{\sum }_{j}\,{{\bf{S}}}_{I}^{j}(t-1)}$$.

The model simulates the build-up of the network as two simultaneous processes with different time scales: the aggregation of new nodes (we call it *node aggregation regime*) and the increase of trade volumes (*weight aggregation regime*). At *t* = 0, the network is just a tiny seed of connected nodes.

**Figure 1:**
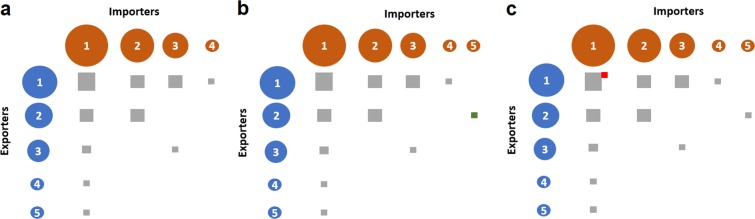
Network formation. (**a**) Weight matrix of a toy network at an early simulation step (*t* < *t*_*F*_). Dot areas are proportional to the global trade of each node, while square areas are proportional to the trade *Exporter*_*i*_ → *Importer*_*j*_. (**b**) *Node aggregation regime*. Importer number 5 appears because of the birth process driven by parameter *λ*_*I*_. It may attach to the existing exporter nodes with a probability proportional to the global trade that each exporter handles at that instant. For this example, the set of Bernouilli experiments yields a new link *Exporter*_2_ → *Importer*_5_ (green square). Its weight is the minimum possible trade volume that we call *token*. (**c**) The *weight aggregation regime* happens from the initial step to the end of the simulation, we have represented it just after the aggregation of *Importer*_5_ for simplicity. A new token attaches to the matrix attracted by a probability that is proportional to the product of the marginal probabilities of *Exporter*_*i*_ and *Importer*_*j*_. Probability is nonzero even for empty cells, this the mechanism that allows the birth of new links. Anyway, most tokens are attracted towards main trade associations, such as the pair *Exporter*_1_ and *Importer*_1_ (red square).

The first process drives the aggregation of nodes of both guilds under the hypothesis of neutrality. That means that the birth probability depends only on the number of nodes of the same guild. Each new node joins the network with a probability that is inverse proportional to the number of nodes in its guild, *N*_*γ*_. A new node may appear with probability *P*_*birth*_*γ*(*t*), where *γ* stands for Exporters or Importers. This value must decrease as the number of nodes raises, and for the sake of simplicity we use the following form:

1 $${P}_{birth}\gamma (t)=\frac{{\lambda }_{\gamma }}{{N}_{\gamma }(k)}$$

where *λ*_*γ*_ is an intrinsic birth rate parameter of guild *γ* and *N*_*γ*_(*t*) is the number of nodes at simulation step *t* (Fig. 1a). Under these assumptions, the time interval between the birth of two consecutive nodes is $\Delta t=\frac{{N}_{\gamma }(t)}{{\lambda }_{\gamma }}$. If the network starts with a seed of size *σ* × *σ* nodes, the formation time is the sum of an arithmetic series:

2 $${\tau }_{\lambda }=\mathop{\sum }\limits_{i=\sigma }^{{N}_{\gamma }}\,\frac{i}{{\lambda }_{\gamma }}=\mathop{\sum }\limits_{i=1}^{{N}_{\gamma }}\,\frac{i}{{\lambda }_{\gamma }}-\mathop{\sum }\limits_{i=1}^{\sigma -1}\,\frac{i}{{\lambda }_{\gamma }}=\frac{{N}_{\gamma }({N}_{\gamma }+1)-{\sigma }^{2}+\sigma }{2{\lambda }_{\gamma }}$$

and then ${\lambda }_{\gamma }=\frac{{N}_{\gamma }({N}_{\gamma }+1)-{\sigma }^{2}+\sigma }{2{\tau }_{\lambda }}$. We set the condition that birth probabilities equal to 1 when the formation process starts. At that time there exists a connected seed of *σ* × *σ* nodes (3 of each class in our practical implementation).

3 $${p}_{\gamma }(0)=\frac{{\lambda }_{\gamma }}{\sigma }=1\Rightarrow {\tau }_{\gamma }=\frac{{N}_{\gamma }({N}_{\gamma }+1)-{\sigma }^{2}+\sigma }{2\sigma }$$

So, the parameter *τ*_*γ*_ depends on the final number of nodes of the network. It changes from year to year for the historical data series, and we perform each simulation experiment using the empirical number of nodes.

The number of nodes is not the same for both guilds due to the applied filtering (see Data Preprocessing section), although figures are quite similar. The formation times of each guild would be different.

4 $${\tau }_{E}=\frac{{N}_{E}({N}_{E}+1)-{\sigma }^{2}+\sigma }{2\sigma },\,{\tau }_{I}=\frac{{N}_{I}({N}_{I}+1)-{\sigma }^{2}+\sigma }{2\sigma }$$

In our model we choose for both guilds the maximum value of *τ*_*E*_ and *τ*_*I*_. This choice has very little impact in the numerical simulation but sets a sharp boundary between both regimes.

During the first, that we call *node aggregation regime*, the number of nodes grows up. Each new node joins the network with a preferential mechanism. Let ${{\bf{W}}}_{EI}^{i,j}(t)$ be the weighted matrix at simulation instant *t*. When an importer [exporter] node appears the probability of attachment to any of the exporter [importer] nodes is ${{\bf{P}}}_{E}^{i}(t)$ [${{\bf{P}}}_{I}^{j}(t)$], as defined above. If the Bernouilli trial is successful, a link with weight 1 will connect both nodes (Fig. 1b). We call this quantum of trade a *token*, and is the minimum possible value of any non-empty cell of the network. The newborn node may tie to more than one existing node of the opposite class, although it is unlikely according to the procedure.

The *weight aggregation regime* starts at *t* = 0 and explains network weight growth. Each token may be seen as a trade chance. At each simulation step a set of new trade chances adds to the ${{\bf{W}}}_{EI}^{i,j}(t)$ matrix, running Bernouilli trials for each cell with probabilities ${{\bf{P}}}_{EI}^{i,j}(t)$.

5 $${{\bf{P}}}_{E}^{i}(t)={{\bf{P}}}_{E}^{i}(t-1){{\bf{P}}}_{I}^{j}(t-1),$$

being ${{\bf{P}}}_{E}^{i}(t-1)=\frac{{{\bf{S}}}_{E}^{i}(t-1)}{{\sum }_{i}\,{{\bf{S}}}_{E}^{i}(t-1)}$, ${{\bf{P}}}_{I}^{j}(t-1)=\frac{{{\bf{S}}}_{I}^{j}(t-1)}{{\sum }_{j}\,{{\bf{S}}}_{I}^{j}(t-1)}$. In case of success one token adds to that cell. By definition, the sum of the elements of ${{\bf{P}}}_{EI}^{i,j}(t)$ is one, so, in average the global trade raises one token per simulation step. Note that an empty cell has a small probability of capturing a trade chance, and by this mechanism the number of links keep on growing even after the network formation instant (from now on, *t*_*F*_, see Fig. 1c). Most trade chances go to existing links, and the time interval between the appearance of new links becomes longer. Eventually, the synthetic matrix reaches the number of links of the empirical matrix and simulation stops at that moment, that we call *t*_*T*_. This aggregation process lasts much longer than the *node aggregation regime* $({t}_{T}\gg {t}_{F})$.

**Figure 2:**
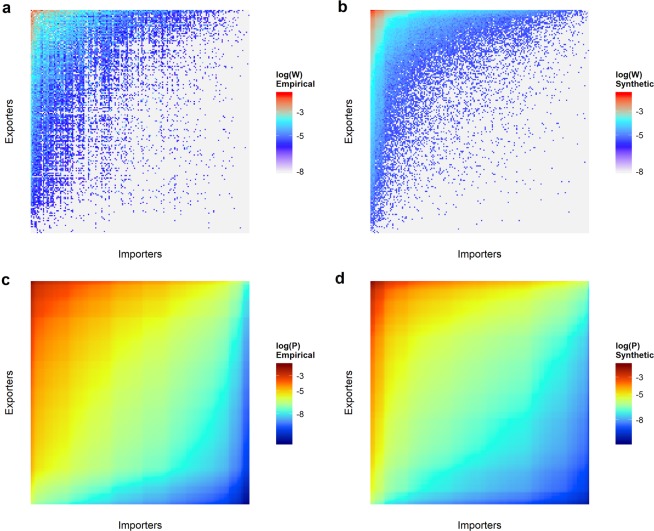
Empirical and synthetic matrixes for year 2015. Normalized weight matrices $\frac{{{\bf{W}}}_{EI}}{{\sum }_{ij}\,{{\bf{W}}}_{EI}}$: (**a**) empirical and (**b**) synthetic at the final instant of the simulation. Below, probability matrices **P**_*EI*_: (**c**) empirical and (**d**) synthetic. Empirical matrixes are plotted using the Observatory of Economic Complexity raw data. Each dot represents a link between one exporter and one importer. Color is assigned according to the normalized weight of that particular yearly trade flow, probabilities are computed as a product of the marginal distributions as explained in the text. Synthetic matrixes are the result of running one simulation experiment with the model, setting just the number of nodes per guild and the number of links equal to those of the empirical matrix.

Finally, as a result of both processes, we obtain the synthetic trade matrix, whose statistical properties may be compared to those of the empirical one (see Fig. 2 and Supplementary Fig. S2c,d). Note that for each year, only three parameters of the empirical network set the final configuration of the synthetic matrix: number of nodes of each guild and number of links of the network.

### Network formation dynamics

As ${{\bf{P}}}_{EI}^{i,j}(t)$ is normalized, the average number of successes of the *N*_*Exp*_(*t*)*N*_*Imp*_(*t*) Bernouilli trials is just 1, and the number of tokens equates the number of simulation steps. This result is straightforward after the formation instant, when the size of the matrix is constant (see Supplementary Information, Fig. S2).

During the *node aggregation regime* the relationship is more complex, as newborn nodes add new links. This figure is of the order of magnitude of *N*_*Exp*_(*t*_*F*_), less than a 10% of the number of tokens at the formation instant and irrelevant compared to the final number of tokens. So, we can make the assumption that at each simulation step the total trade volume grows up by one token, and the number of tokens equates simulation time.

The number of links follows a quadratic law over time. A new link appears when the token falls in an empty cell of ${{\bf{W}}}_{EI}^{i,j}$, otherwise the overall strength increases but the number of links remains constant. The probability of this event is ${P}_{empty}(t)={\sum }_{ij|{{\bf{W}}}_{EI}^{i,j}(t-1)=0}\,{{\bf{P}}}_{EI}^{i,j}(t-1)$, while the token falls in a non-empty cell with probability *P*_*full*_(*t*) = 1 − *P*_*empty*_(*t*).

**Figure 3:**
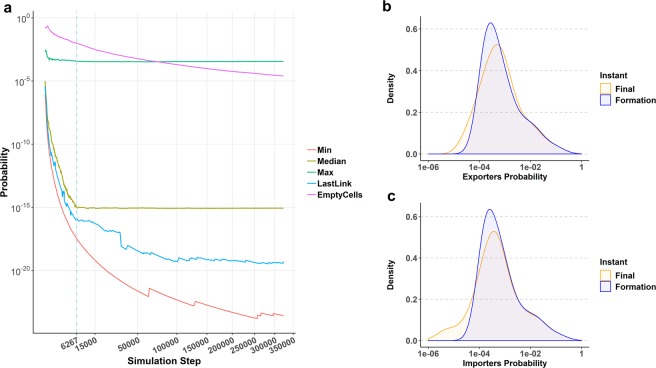
Evolution of the values of the synthetic probability matrix for year 2008. (**a**) Minimum and maximum values of ${{\bf{P}}}_{EI}^{i,j}(t)$ and its median over the simulation time. In addition, we plot the sum of probabilities of all empty weight cells *P*_*empty*_(*t*), that means all possible combinations Exporter/Importer without trade at that instant. Finally, we add the probability value for the last cell where a new link has appeared, (*LastLink* in the legend). The dashed blue line marks the formation instant. (**b**,**c**) Marginal probability distributions **P**_*E*_(*t*) and **P**_*I*_(*t*) at *t*_*F*_ and *t*_*T*_. Note that their shapes evolve towards log-normality.

Probabilities are not constant over the simulation spain. Figure 3a shows some interesting properties to simplify the analysis. The maximum value of ${{\bf{P}}}_{EI}^{i,j}(t)$ corresponds to the strongest trade flow of the matrix. This Exporter/Importer pair attracts the majority of tokens and the probability remains almost constant over time. As trade volume tends to attach to existing links, the sum of probabilities of the set of empty cells decreases, an so the minimum value of the matrix. Two interesting values are those of the probability of the cell where the last link of the simulation appeared before any given simulation instant and the median of the probability matrix. The plot shows a sharp contrast before and after *t*_*F*_. During the node aggregation regime, each attached node comes with new empty cells that will have low probability values in contrast with those of populated cells. After *t*_*T*_, matrix size is fixed, no new empty cells may appear, they only may evolve to populated cells. The probability of the last filled cell remains almost constant for the last two thirds of the simulation time, as well. The probability matrix ${{\bf{P}}}_{EI}^{i,j}(t)$ plays in *Synthrade* the same role of the growth distribution in Ijiri and Simon’s model, as ict acts as the network growth driver. It is strongly right-skewed, but the median remains almost constant, and this property yields log-normality in synthetic trade degree and strentgth.

As a consequence of this evolution, time between arrival of new links does not grow linearly. Assume that the global strength value is *m*, and the number of links *l*, when the first link appears after *t*_*F*_. As the variation of *P*_*empty*_(*t*) is so slow, the addition of new links may be viewed as a Bernouilli process. The mean time between the appearance of link *l* and link *l* + 1 is 1/*P*_*empty*_(*t*). During that interval, the non-empty cells attract a quantity *m* of tokens, plus the token that falls in the empty cell, so *t*_*l*+1_ − *t*_*l*_ = *m* + 1. To add a second link, the network adds *m* + 1 tokens to the non-empty cells, plus the token of the new link, so *t*_*l*+2_ − *t*_*l*+1_ = *m* + 2. The time to add *L* links is given by the formula:

6 $${t}_{l+L}-{t}_{l}=\mathop{\sum }\limits_{j=1}^{L}\,(m+j)=\frac{L(m+1+m+L)}{2}=\frac{{L}^{2}+(2m+1)L}{2}$$

So, for a linear growth of *L* links, the strength obeys a quadratic law, and the simulation time is *O*(*n*^2^) (see Supplementary Information, Fig. S2a). During the *node aggregation regime*, the matrix size is not constant and the hypothesis of almost stationary probabilities is not longer valid. Anyway, the number of links and nodes of both guilds follow quadratic patterns as well (see Supplementary Fig. S2b).

### Strength and degree distributions

The plots of empirical networks suggested that strength and degree follow a power law *s* ∝ *k*^*β*^ (see Supplementary Information, Fig. S4). To estimate the slope *β*, we use the cumulative distribution^27^ and fit a log-log regression line to get *β* + 1.

7 $${\int }_{0}^{k}s(k^{\prime} )dk^{\prime} ={\int }_{0}^{k}C{k}^{^{\prime} \beta }dk^{\prime} =\frac{C}{\beta +1}{k}^{\beta +1}$$

**Figure 4:**
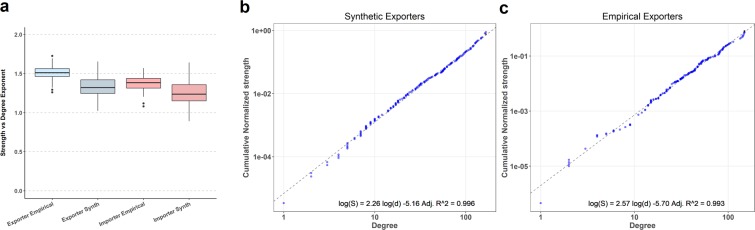
Strength vs Degree slope. (**a**) Distributions of *β* exponent for 56 empirical networks and 1680 synthetic networks. (**b**,**c**) Estimation for an individual simulation of year 1998 using linear regression.

As Barrat *et al*. showed^28^, in the lack of correlations between the weight of the links and the degree of the nodes, the strength of a node is simply proportional to its degree, yielding an exponent 1, consequently, strength and degree provide the same information on the system. In our study, the values of *β* are always above 1 for empirical and synthetic networks (Fig. 4 and Supplementary Information, Fig. S3). The strength of the most connected nodes tends to have a value higher than the corresponding to a random process of weights. In other terms, main exporters (importers) attract more volume than if trade flow were just proportional to the number of countries they trade with.

**Figure 5:**
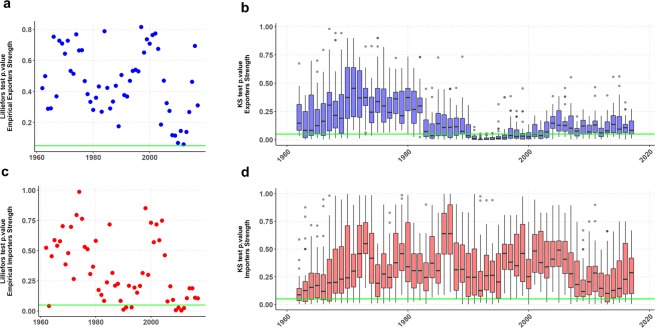
Goodness of fit tests. (**a**,**c**) Normality test of *W*_*EI*_ empirical networks. The green lines are located at p = 0.05. (**b**,**d**) Kolmogorov-Smirnov distance test among *W*_*EI*_ empirical and synthetic networks. Each whisker plot represents the p-values of the K-S distances between the empirical network and each one of the 30 synthetic *W*_*EI*_(*t*_*T*_) matrixes we build for that particular year.

The distribution of the empirical weight matrices tends towards a log-normal, and the synthetic ones closely follow their shapes. To test this hypothesis we perform a goodness of fit analysis of the logarithmic values of the empirical strength distributions of both Exporters and Importers, with the Lilliefors test. Setting a *p*-*value* of 0.05, the hypothesis of non-normality is rejected for 8 out of 56 importer distributions and none of exporter distributions (Fig. 5a,b, see Supplementary Information, Table S1).

**Figure 6:**
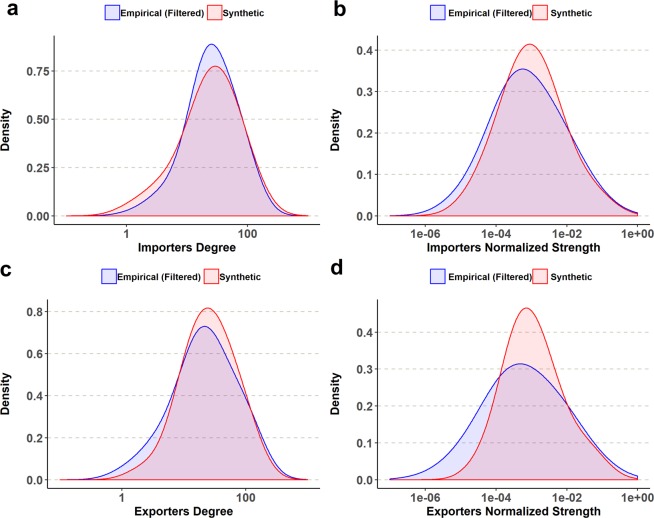
Density plots for year 1986. (**a**,**c**) Degree distributions. (**b**,**d**) Normalized strength distributions.

To measure the shape similarity between the normalized empirical strength distributions and their synthetic counterparts we set the following procedure. For each year, we build 30 synthetic networks, running 30 independent experiments with the same three model parameters values (number of importers and exporters and number of links) of the empirical network. Then, we perform a Kolmogorov-Smirnov distance test between the empirical matrix and each one of the synthetic matrixes. According to these results the model mimics quite well the exporter distributions. Boxplots in Fig. 5c,d are the set of K-S test values for the marginal exporter and importers distributions of each year. The horizontal green line, is set at p = 0.05. For this test, the null hypothesis is that the empirical and the synthetic distributions are different. The hypothesis may be rejected for almost all exporter distributions. On the other hand, the null hypothesis of dissimilarity can’t be rejected for importers during the 1990’s, when connectance dropped in the aftermath of the dissolution of the Soviet Union. Figure 6 shows, as a typical example, a visual comparison of degree and strength distributions for year 1986.

Marginal probability distributions **P**_*E*_(*t*) and **P**_*I*_(*t*) are strongly right-skewed at *t*_*T*_ (Fig. 3b,c). Their shape tends towards log-normality as the simulation enters the *weight aggregation regime* and network size remains constant. The explanation is that empty cells have more choices over time of being populated, and from that instant on, they join the multiplicative process. Despite the fact that their strength is small, the distribution is more even because of the increase of the fraction of non-empty cells. The same pattern is observed for the probability matrix **P**_*EI*_.

Log-normality of the strength and degree distributions is a result of the multiplicative process^29^. Let ${W}_{E}^{j}({t}_{F})$ be the strength of exporter *j* at *t*_*F*_, we write it ${W}_{E}^{j}(0)$ for the sake of simplicity. One simulation step later ${W}_{E}^{j}(1)={W}_{E}^{j}(0)(1+{X}_{E}^{j}(0))$ where ${X}_{E}^{j}(0)$ is a Bernouilli random variable with probability ${P}_{E}^{j}(0)$. After *m* simulation steps the strength will be:

8 $${W}_{E}^{j}(m)={W}_{E}^{j}(0)(1+{X}_{E}^{j}(0))(1+{X}_{E}^{j}(1))\ldots (1+{X}_{E}^{j}(m))$$

Taking natural logarithms, the expression becomes:

9 $${\log }({W}_{E}^{j}(m))=\,{\log }({W}_{E}^{j}(0))+\mathop{\sum }\limits_{k=0}^{m}\,{\log }(1+{X}_{E}^{j}(k))$$

Variables ${\log }(1+{X}_{E}^{j}(k))$ are independent, as each token addition experiment only depends on the values of *P*_*EI*_ at simulation step *t*. As the average value of ${X}_{E}^{j}(k)\ll 1$, then ${\log }(1+{X}_{E}^{j}(k))$ is also quite smaller than 1. The standard deviation of a sum of independent random variables is the sum of the standard deviations, and so, the value of $Var({\log }({W}_{E}^{j}(m)))$ is greater than 1 and finite. It holds also that $Var({\log }(1+{X}_{E}^{j}(k)))\ll Var({\log }({W}_{E}^{j}(m)))$. The Lindeberg–Levy conditions are met and the distribution of *W*_*E*_ approaches a log-normal.

## Discussion

In this work we have proposed a generative model (that we call *Synthrade*) to build synthetic bipartite networks that closely resemble the statistical properties of size distributions of the historical series. The bipartite hypothesis is a valid alternative to the time-evolving directed network model^16^ and more expressive than unidirected representations^17,26^.

Although the World Trade Network is a very complex system that emerges from the aggregation of multiple choices at different levels (individuals, companies, regulatory bodies, and so on) *Synthrade* reproduces the statistical properties of the recorded annual trade data working on two principles: preferential attachment and network strength growth by product of probabilities. Trade flows are driven by the relative strength of exporter and importer nodes, any new trade chance attaches to a cell of the bipartite trade matrix with a probability that is the product of the marginal probabilities of both edges.

Network strength grows by probability, though, this fact does not explain initial network formation. The preferential attachment mechanism that acts before *t*_*F*_ is the basis of the observed strength heterogeneity in empirical data. *Synthrade* is naive in the sense that it loses memory from year to year and trade agreements and contracts do not work like that. The explanation on how this relates to real world trade could be the following. Node aggregation is a very fast process compared to the total simulation time. Nodes that arrive early enjoy higher probability of capturing new trade chances. In addition, aggregation speed decreases over simulation time, so first comer nodes will be the main trade attractors, while minor players will enter the network later. The parameter *λ* governs that speed. Simulation time should not be regarded as a proxy of real time. Simulation steps only represent the arrival of a new trade token. With this idea in mind, the node aggregation regime is not some small period of each January, but an almost instantaneous and convenient mechanism to generate heterogeneity.

Furthermore, the relationship degree-strength of the synthetic networks follows a power-law, as it happens in the empirical series. Any incoming node connects to major sellers (or buyers) as a first choice and links appear as time goes by during the network build-up period, but at a diminishing pace. Once the network has reached its maximum size, incoming trade chances are the only mechanism to create new links. As noted by Garlaschelli *et al*.^16^, the WTN may be explained by the *network fitness model*^18^ using GDP as the *hidden variable*. In *Synthrade*, the force that fosters the birth of new links through mutual benefit is proportional to the product of the strengths of both nodes. As new trade opportunities tend to join the main flows Exporter-Importer, dominant nodes attract more and more volume. The exponent of the degree-strength distribution shows that they are over-trading, while smaller countries strive to play their role in this global network^28^.

As trade chances (tokens) have a small and constant weight, their power to modify the underlying probability matrix weakens over time. Weight and probability matrixes tend towards quasi-stationary states and the long-term effect of this behaviour is a multiplicative growth process that pushes the strength and degree distributions towards a log-normal.

*Synthrade* is a remarkable simple model that does not take into account many constraints, as distance, trade agreements, tariffs or competitive advantages^30^. In particular, it ignores factors such as distance or some GDP proxy, unlike the family of gravity bilateral trade models^21,31^. It works on a global scale trying to reproduce the network degree sequences that are highly informative instead^1^. On the other hand, it does not require to know in advance that a trade link exist between a pair of countries. A recent advance by Almog, Squartini and Garlaschelli has reconciled the accuracy of gravity models to estimate the intensity of trade flows with its inability to discover network topology, using GDP as a fitness parameter^32^. *Synthrade* does not include macroeconomic information, just the interaction matrixes data because they are enough to reach its goal of reproducing trade volume topological properties.

This set of simplifications opens the way to further improvements. The node aggregation regime could benefit of the addition of rules of affinity or prohibitions^33^. Similarly, the probability matrix might include some modulation function to model geographical distance^34,35^. Finally, it would be interesting to check if degree and strength follow similar patterns when trade is restricted to some particular good or category of goods, and if so, the bipartite WTN shows fractal properties^36^.

## Methods

### Data preprocessing

We work with the historical data series from 1962 to 2017, available at The Observatory of Economic Complexity^37^. For each year there is one file that holds per line the ISO code of the exporter country, the ISO code of the importer, a category key of the traded merchandise and the yearly amount in dollars. For this paper we have worked with global trade, just adding the monetary value of all exchanged goods between each pair Exporter/Importer. With this information we build the empirical weight matrix (Fig. 2a).

Quality issues arise from two different product classification systems (SITC and HC), two different statistical sources (from 1962 to 2000^38^ and from 2001 to 2017^39^), two different harmonization approaches^38,40^ and geopolitical events^41,42^ during such a long span. In addition, trade networks are strongly meshed (average connectance is 0.49) because of the existence of very weak links. To overcome these problems we apply two mild mitigation strategies.

There is a step decrease of connectance (ratio of existing links over total possible links) after 1983, because of the inclusion of 38 new country codes. Almost all of them are island countries, their data were aggregated in supranational entities before that year. As their contribution to the global trade volume is tiny, we drop those nodes for our analysis from 1983 to 2017.

After 1991, the reshape of the political maps of Eastern Europe and Central Asia produced a second fall of connectance event. It is impossible to follow the same approach because in this case the resulting political entities are as relevant as the Russian Federation. We keep former and new countries as separated entities for the global analysis (see Supplementary Information, Fig. S6).

To avoid the strongly meshed condition, a common solution is removing those links that account for a small fraction of the global trade^22,43^. We just drop those that fall within the lower 0.1% of total trade distribution for each year. The effect of the filtering is irrelevant for weight, as 99.9% of trade volume is kept, but not for degree^44,45^, because many tiny interactions are lost. As we are studying the aggregated figures of trade, we think this information loss is not critical for our analysis, but it is important to inform the reader about this issue.

After these preprocessing steps we create the weighted interaction matrix. It has one row per exporter country and one column per importer. We fill each cell with the global trade from exporter to importer or set it zero if there were no recorded trade or the volume falls below the filtering threshold. From this matrix, we obtain the number of exporters an importers and the number of links, the parameters that govern the synthetic network dynamics.

### R packages

The statistical analyzes and their respective graphs have been carried out with different R package: Lilliefors and Kolmogorov-Smirnov tests with nortest^46^ and densities estimation are plotted with the ggplot2^47^ (geom_density() with bandwith adjustment = 2).

## Supplementary information

A stochastic generative model of the World Trade Network (Supplementary Material)

## Data Availability

Code and data are available at https://zenodo.org/badge/latestdoi/120930457. Reproducibility instructions are detailed in the README.md file.
