# Supplementary material for: A stochastic generative model of the World Trade Network
Source: Sci Rep. 2019 Dec 6;9:18539. doi: 10.1038/s41598-019-54979-1 (PMC6897986; doi:10.1038/s41598-019-54979-1)
Supplement: Supplementary file 1 — A stochastic generative model of the World Trade Network (Supplementary Material) [file 41598_2019_54979_MOESM1_ESM.pdf]

A stochastic generative model of the World Trade Network (Supplementary Material)

Javier García-Algarra[a], Mary Luz Mouronte-López[b], Javier Galeano[c]

[a] *Department of Engineering, Centro Universitario de Tecnología y Arte Digital, Las Rozas, Spain*
[b] *Department of Computer Science, Universidad Francisco de Vitoria, Pozuelo de Alarcón, Spain*
[c] *Complex Systems Group, Universidad Politécnica de Madrid, Madrid, Spain*

S1. Figures

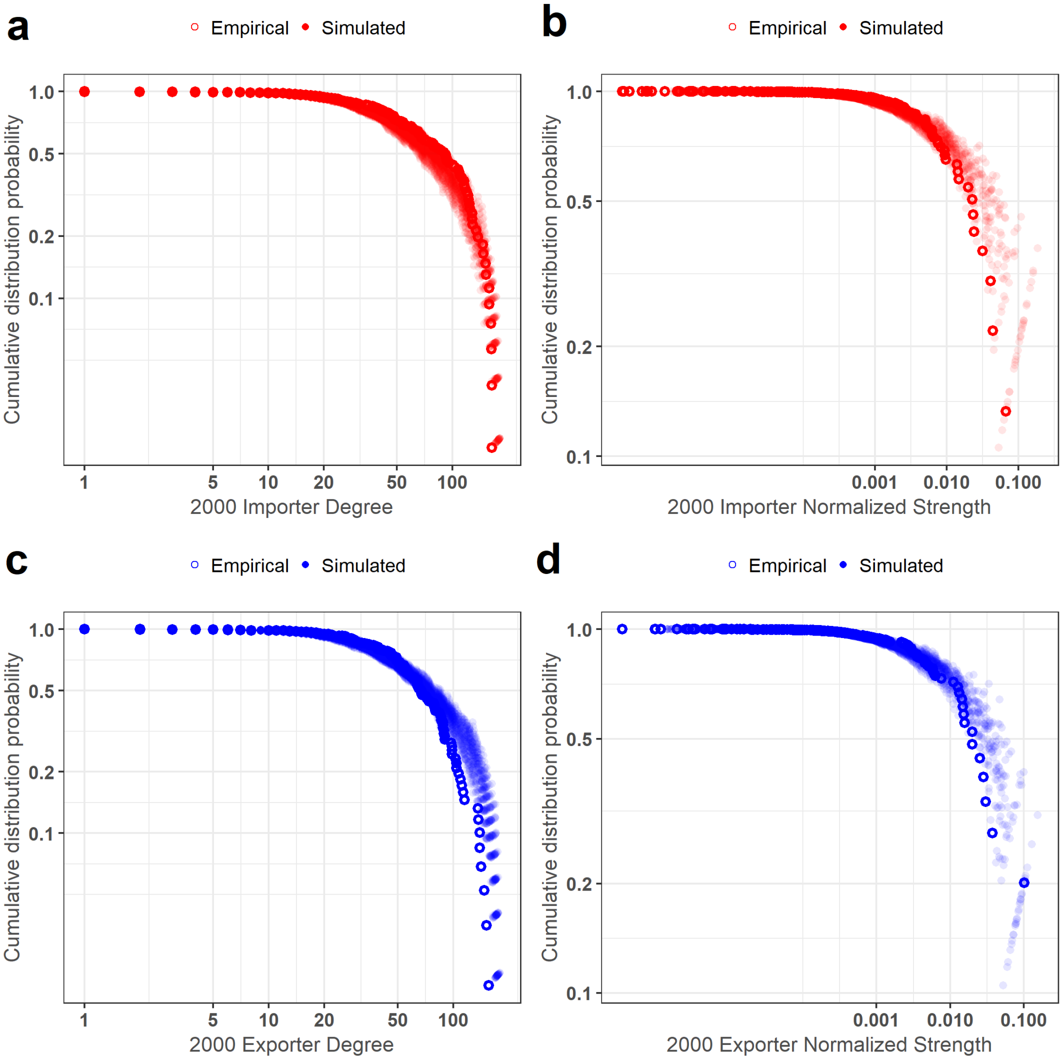

Figure S1: **Cumulative degree and strength distributions for year 2000**. (a, b) Exporter nodes. (c, d) Importer nodes. Superimposed distribution of empirical values (circumferences) on 30 synthetic experiments (cloud of dots). It is clear that they do not show the characteristic linear pattern of a power-law.

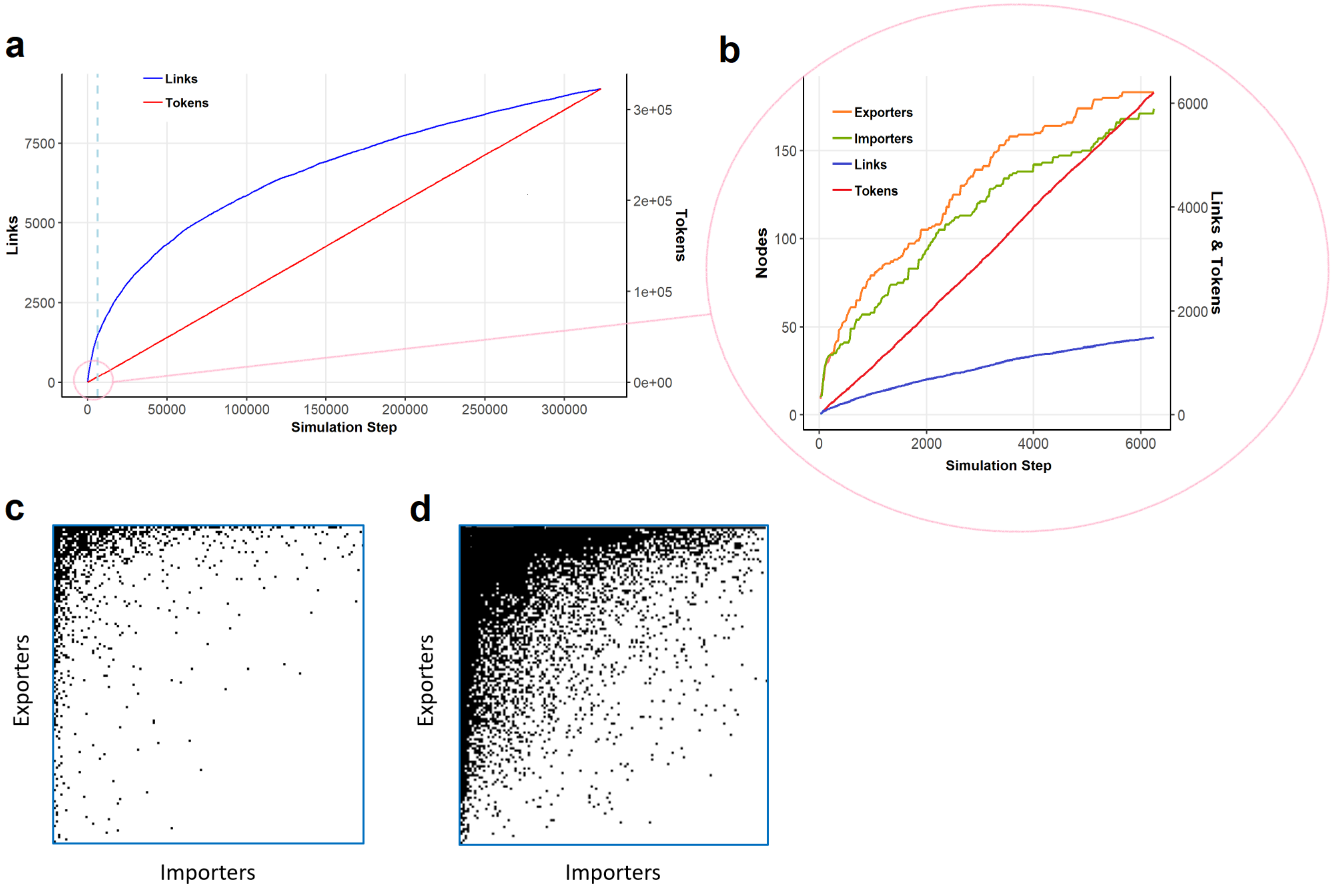

Figure S2: **Growth dynamics**. (a) Links and tokens evolution over simulation time of the synthetic network for year 2008. (b) Zoom of the node aggregation regime period, showing the evolution of nodes as well. (c) Unweighted synthetic network at t_F for the year 1962, with 138 exporters, 141 importers and 1109 links. (d) The same synthetic network at t_T with 4696 links. For this year t_T is around 18 times t_F.

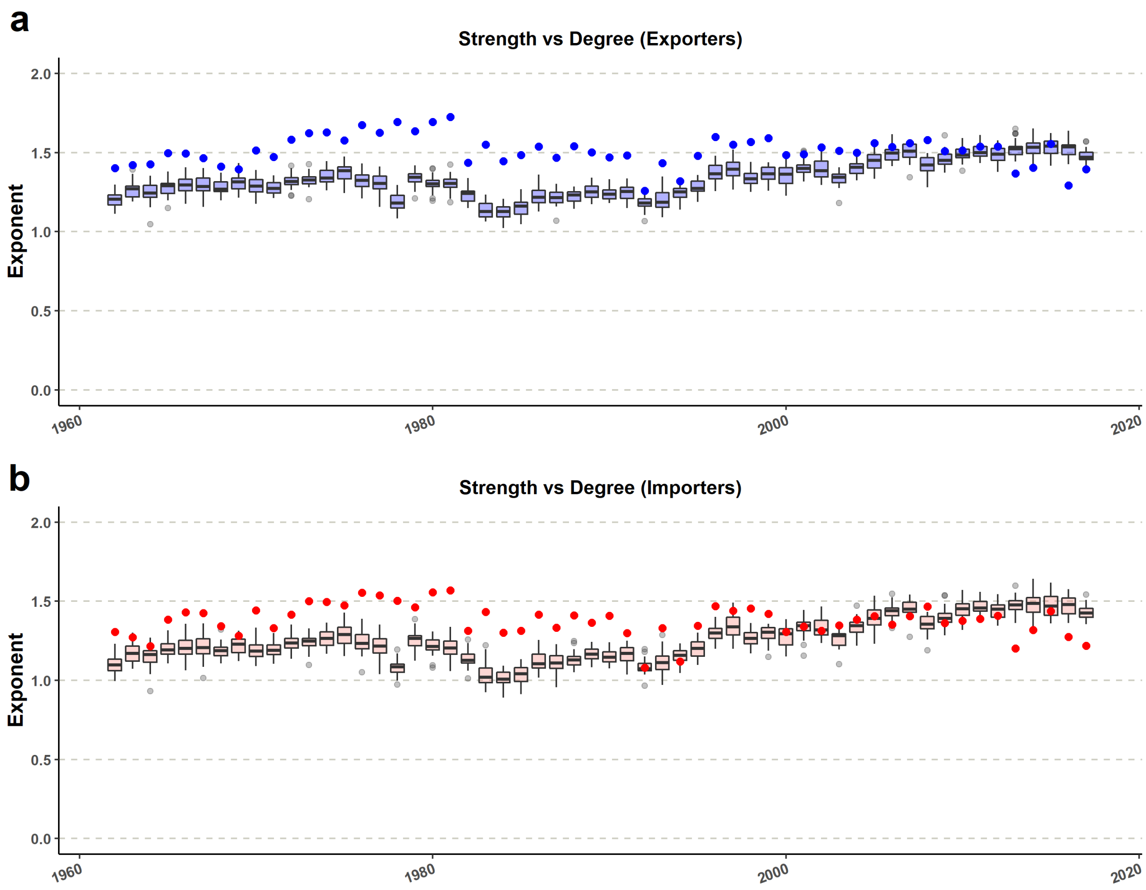

Figure S3: **Strength-Degree slopes**. Empirical values as dots and synthetic experiments as boxplots for Exporters (a) and Importers (b).

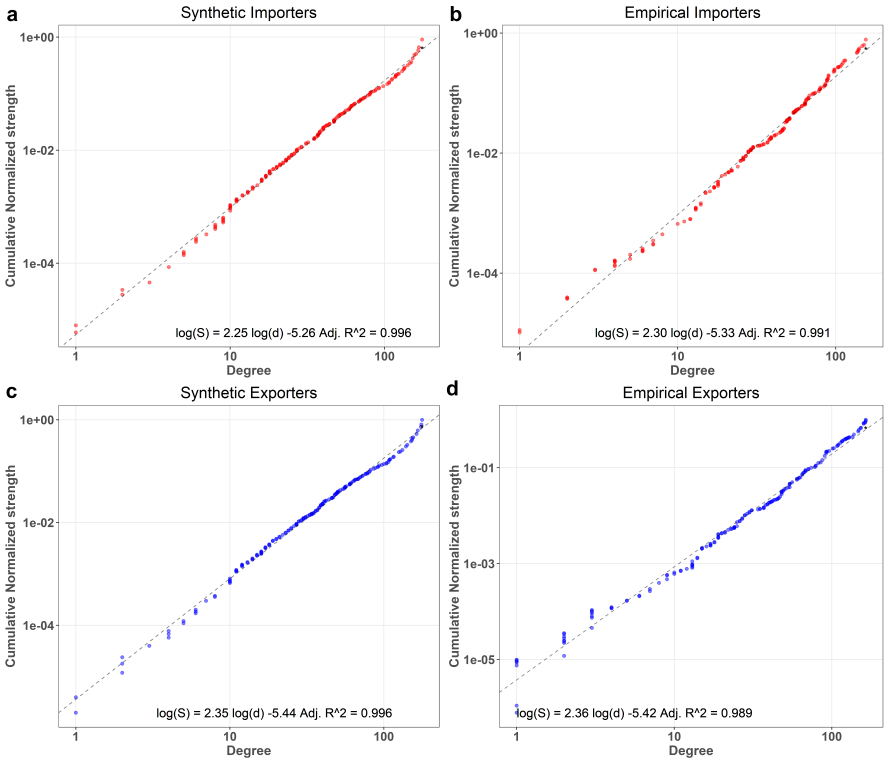

Figure S4: **Strength-Degree slope for year 2000**. (a, c) Synthetic matrixes. (b, d) Empirical matrixes.

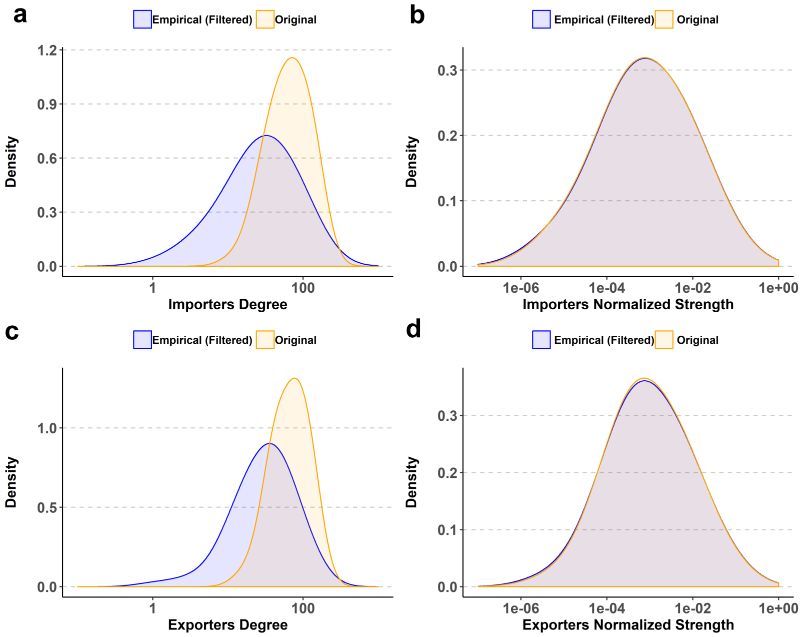

Figure S5: **Distributions for year 1980**. (a, c) Degree distributions for clean and filtered matrixes (see Fig. S6 for explanation). (b, d) Normalized strength distributions.

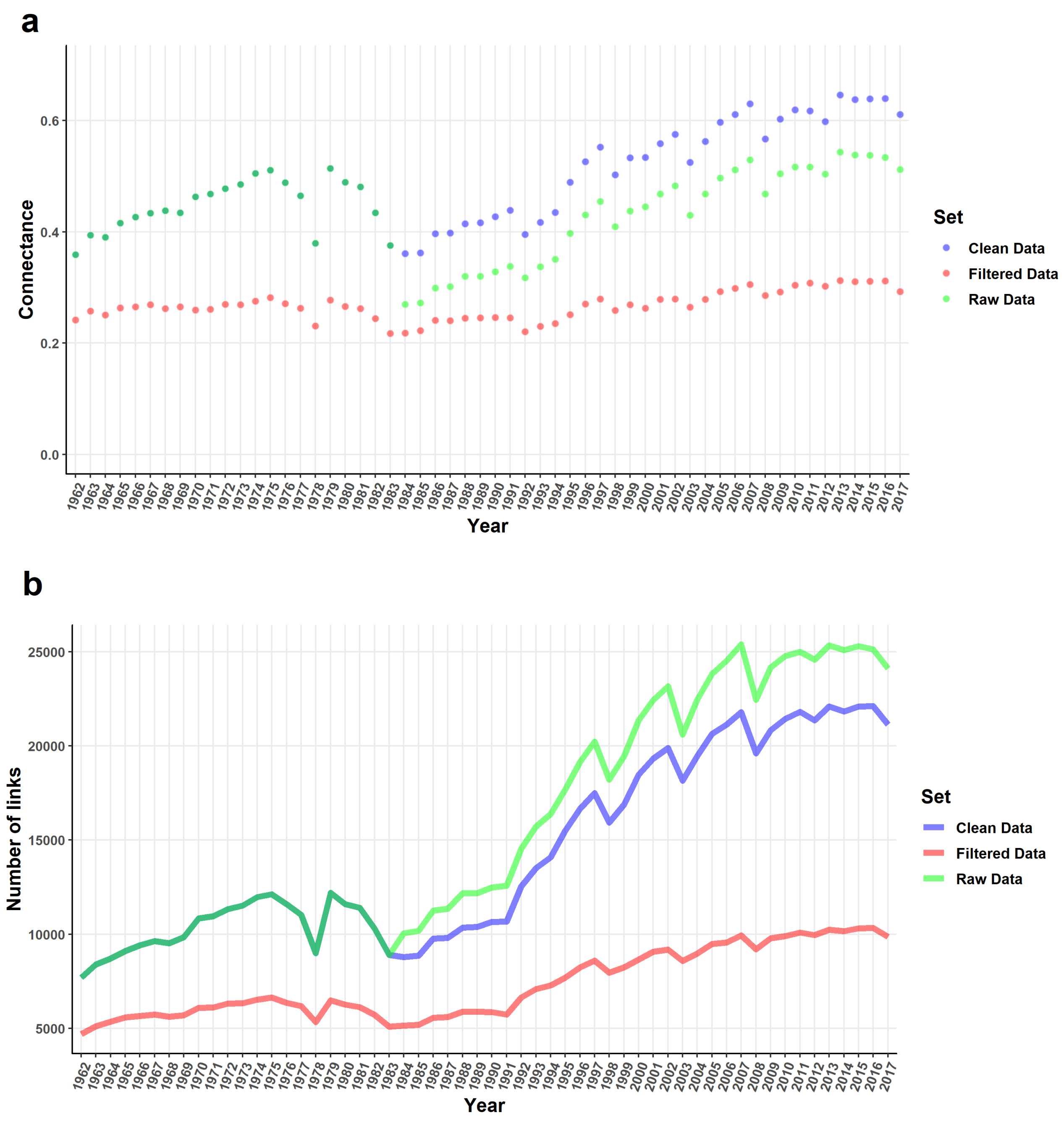

Figure S6: **Connectance and number of links of historical series**. (a) Connectance: raw data (average connectance 0.49), clean data (average connectance 0.43) and filtered data (average connectance 0.23). Clean data series is identical to raw data series before 1984, when 38 country codes of microstates were added. For this study, they are dropped (AIA,AND,ANT,ATG,BRN,BTN,CCK,COK,COM,CPV,CXR,CYM,DMA,ESH,FRO,GRD,IOT,LCA,MDV,MSR,MTQ,NFK, NIU,NRU, PCI,PCN,PYF,REU, SLB,STP,TCA,TON,TUV,VCT,VGB,VUT,WLF,YEM). (b) Number of links. Filtered data ammounts 99.9% of yearly global trade.

S2. Tables

Year	Empirical Exporters	Empirical Importers
1962	0.4210	0.5238
1963	0.4986	0.0419
1964	0.2878	0.4529
1965	0.2904	0.5860
1966	0.7529	0.5399
1967	0.3681	0.5772
1968	0.7267	0.7022
1969	0.7084	0.3853
1970	0.6434	0.4793
1971	0.7270	0.6963
1972	0.5325	0.2645
1973	0.5141	0.7946
1974	0.7684	0.9869
1975	0.6641	0.7628
1976	0.6664	0.5300
1977	0.4665	0.5114
1978	0.3831	0.3064
1979	0.3325	0.3673
1980	0.2804	0.5806
1981	0.3588	0.1742
1982	0.4316	0.1331
1983	0.2690	0.0843
1984	0.7884	0.2366
1985	0.4232	0.7168
1986	0.2902	0.3154
1987	0.3347	0.2104
1988	0.4364	0.2249
1989	0.1753	0.0832
1990	0.5060	0.0108
1991	0.3764	0.0303
1992	0.3669	0.2085
1993	0.4687	0.0302
1994	0.5324	0.1932
1995	0.5392	0.1515
1996	0.5290	0.2075
1997	0.8153	0.3145
1998	0.6508	0.8514
1999	0.7358	0.2995
2000	0.7079	0.7307
2001	0.7633	0.5696
2002	0.7739	0.7168
2003	0.6742	0.5852
2004	0.1860	0.7478
2005	0.4693	0.4616
2006	0.3247	0.0786
2007	0.2747	0.2037
2008	0.1177	0.0915
2009	0.1146	0.0065
2010	0.0673	0.0276
2011	0.1453	0.0045
2012	0.0579	0.0241
2013	0.1383	0.1064
2014	0.2666	0.1879
2015	0.4619	0.1873
2016	0.6932	0.1094
2017	0.3100	0.1053

Table S1: **Log-normality test for empirical strength distributions**. The null hypohtesis is that the each yearly ditribution follows a log-normal distribution. The Lilliefors test fails to reject the log-normality hypothesis for all exporters and 48 out of 56 importers with a p-value of 0.05

.
